# 1-[(*Z*)-2-Phenyl­hydrazin-1-yl­idene]-1-(piperidin-1-yl)propan-2-one

**DOI:** 10.1107/S1600536811029680

**Published:** 2011-07-30

**Authors:** Hatem A. Abdel-Aziz, Seik Weng Ng, Edward R. T. Tiekink

**Affiliations:** a Department of Pharmaceutical Chemistry, College of Pharmacy, King Saud University, Riyadh 11451, Saudi Arabia; b Department of Chemistry, University of Malaya, 50603 Kuala Lumpur, Malaysia; c Chemistry Department, Faculty of Science, King Abdulaziz University, PO Box 80203 Jeddah, Saudi Arabia

## Abstract

A *Z* configuration about the imine bond [1.3025 (18) Å] in the title compound, C_14_H_19_N_3_O, allows for the formation of an intra­moleclar N—H⋯N hydrogen bond between the hydrazone H and piperidine N atoms; the carbonyl group is disposed to lie over the piperidine residue, which is in a chair form. A twist between the terminal benzene ring and the hydrazine residue is seen [N—N—C—C torsion angle = 163.81 (12)°]. Helical supra­molecular chains along the *c* axis mediated by N—H⋯O hydrogen bonds are the most prominent feature of the crystal packing. The chains are connected into layers lying in the *ac* plane by weak C—H⋯π contacts involving two methyl­ene H atoms and an adjacent benzene ring.

## Related literature

For background to the biological activity of amidrazones, see: Frohberg *et al.* (2006 ▶); Abdel-Aziz & Mekawey (2009 ▶); Abdel-Aziz *et al.* (2010 ▶). For the synthesis, see: Frohberg *et al.* (1995 ▶).
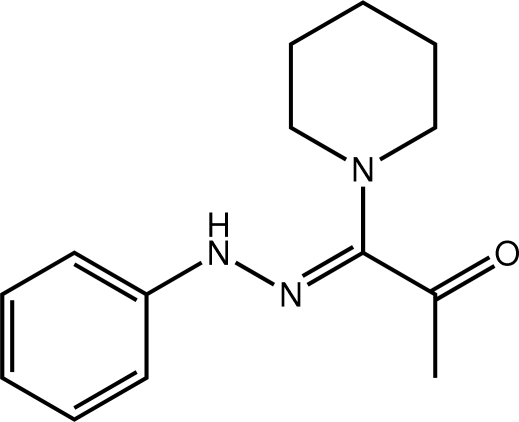


## Experimental

### 

#### Crystal data

C_14_H_19_N_3_O
*M* *_r_* = 245.32
Orthorhombic, 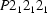

*a* = 9.1195 (2) Å
*b* = 11.9614 (2) Å
*c* = 12.0393 (2) Å
*V* = 1313.27 (4) Å^3^
*Z* = 4
Cu *K*α radiation
μ = 0.64 mm^−1^
*T* = 100 K
0.25 × 0.10 × 0.05 mm

#### Data collection

Agilent SuperNova Dual diffractometer with an Atlas detector
Absorption correction: multi-scan (*CrysAlis PRO*; Agilent, 2010 ▶) *T*_min_ = 0.857, *T*_max_ = 0.969
5335 measured reflections
2589 independent reflections
2482 reflections with *I* > 2σ(*I*)
*R*_int_ = 0.021

#### Refinement

*R*[*F* ^2^ > 2σ(*F* ^2^)] = 0.033
*wR*(*F* ^2^) = 0.080
*S* = 1.05
2589 reflections
168 parameters
H atoms treated by a mixture of independent and constrained refinement
Δρ_max_ = 0.12 e Å^−3^
Δρ_min_ = −0.20 e Å^−3^
Absolute structure: Flack (1983 ▶), 1077 Friedel pairs
Flack parameter: −0.1 (3)

### 

Data collection: *CrysAlis PRO* (Agilent, 2010 ▶); cell refinement: *CrysAlis PRO*; data reduction: *CrysAlis PRO*; program(s) used to solve structure: *SHELXS97* (Sheldrick, 2008 ▶); program(s) used to refine structure: *SHELXL97* (Sheldrick, 2008 ▶); molecular graphics: *ORTEP-3* (Farrugia, 1997 ▶) and *DIAMOND* (Brandenburg, 2006 ▶); software used to prepare material for publication: *publCIF* (Westrip, 2010 ▶).

## Supplementary Material

Crystal structure: contains datablock(s) global, I. DOI: 10.1107/S1600536811029680/hb6327sup1.cif

Structure factors: contains datablock(s) I. DOI: 10.1107/S1600536811029680/hb6327Isup2.hkl

Supplementary material file. DOI: 10.1107/S1600536811029680/hb6327Isup3.cml

Additional supplementary materials:  crystallographic information; 3D view; checkCIF report

## Figures and Tables

**Table 1:** Hydrogen-bond geometry (Å, °) *Cg*1 is the centroid of the C9–C14 benzene ring.

*D*—H⋯*A*	*D*—H	H⋯*A*	*D*⋯*A*	*D*—H⋯*A*
N3—H3⋯O1^i^	0.88 (2)	2.47 (2)	3.2317 (15)	145.6 (17)
N3—H3⋯N1	0.88 (2)	2.242 (19)	2.6340 (16)	106.8 (15)
C2—H2b⋯*Cg*1^ii^	0.99	2.77	3.5535 (16)	137
C3—H3a⋯*Cg*1^iii^	0.99	2.98	3.9473 (16)	167

Symmetry codes: (i) 
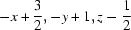
; (ii) 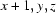; (iii) 
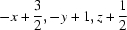
.

**Fig. 1.**
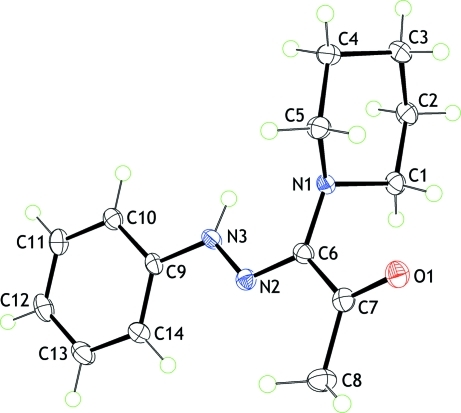
The molecular structure of (I) showing displacement ellipsoids at the 50% probability level.

**Fig. 2.**
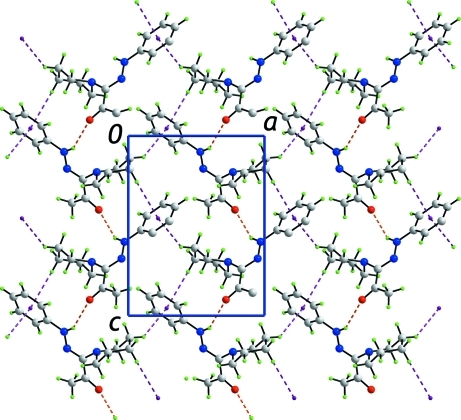
Supramolecular array aligned in the ac plane in (I) mediated by N—H···O and C—H···π interactions shown as orange and purple dashed lines, respectively.

## supplementary crystallographic information

### Comment

The title compound (I) and related amidrazone derivatives are known to possess biological activity (Frohberg *et al.*, 2006), the motivation for on-going studies in this area (Abdel-Aziz & Mekawey, 2009; Abdel-Aziz *et al.*, 2010). In the molecule of (I), Fig. 1, the configuration about the imine N2═C6 bond [1.3025 (18) Å] is *Z*. This places the hydrazone-N3—H in close proximity to the piperidinyl-N1 enabling the formation of an intramolecular N—H···N hydrogen bond (Table 1). The carbonyl group is disposed to lie over the piperidinyl group which adopts a chair conformation. The benzene group is twisted out of the plane through the hydrazine residue to which it is connected as seen in the value of the N2—N3—C9—C10 torsion angle of 163.81 (12) °. The piperidinyl group is disposed to be almost normal to the rest of the molecule so that the dihedral plane formed through its least-squares plane and that through the O1,N2,N3,C6,C7,C9 atoms is 85.26 (6) °.

In the crystal, molecules are connected into a helical supramolecular chain mediated by N—H···O hydrogen bonds (Table 1). Chains are orientated along the *c* axis and are connected into a supramolecular array in the *ac* plane by C—H···π interactions involving methylene-H atoms associated with the bifurcated benzene ring (Table 1 and Fig. 2).

### Experimental

This compound was prepared by the reaction of 2-oxo-*N*'-phenylpropanehydrazonoyl chloride with piperidine (Frohberg *et al.*, 1995). The yellow prisms of (I) were isolated from its ethanol solution by slow evaporation at room temperature.

### Refinement

Carbon-bound H-atoms were placed in calculated positions [C—H 0.95 to 0.99 Å, *U*_iso_(H) 1.2 to 1.5*U*_eq_(C)] and were included in the refinement in the riding model approximation. The amino-H atom was located in a difference Fourier map, and subsequently refined freely.

### Crystal data

**Table:**

C_14_H_19_N_3_O	*F*(000) = 528
*M**_r_* = 245.32	*D*_x_ = 1.241 Mg m^−^^3^
Orthorhombic, *P*2_1_2_1_2_1_	Cu *K*α radiation, λ = 1.5418 Å
Hall symbol: P 2ac 2ab	Cell parameters from 3524 reflections
*a* = 9.1195 (2) Å	θ = 3.7–73.9°
*b* = 11.9614 (2) Å	µ = 0.64 mm^−^^1^
*c* = 12.0393 (2) Å	*T* = 100 K
*V* = 1313.27 (4) Å^3^	Prism, yellow
*Z* = 4	0.25 × 0.10 × 0.05 mm

### Data collection

**Table:**

Agilent SuperNova Dual diffractometer with an Atlas detector	2589 independent reflections
Radiation source: SuperNova (Cu) X-ray Source	2482 reflections with *I* > 2σ(*I*)
mirror	*R*_int_ = 0.021
Detector resolution: 10.4041 pixels mm^-1^	θ_max_ = 74.1°, θ_min_ = 5.2°
ω scans	*h* = −11→7
Absorption correction: multi-scan (*CrysAlis PRO*; Agilent, 2010)	*k* = −14→11
*T*_min_ = 0.857, *T*_max_ = 0.969	*l* = −15→13
5335 measured reflections	

### Refinement

**Table:**

Refinement on *F*^2^	Secondary atom site location: difference Fourier map
Least-squares matrix: full	Hydrogen site location: inferred from neighbouring sites
*R*[*F*^2^ > 2σ(*F*^2^)] = 0.033	H atoms treated by a mixture of independent and constrained refinement
*wR*(*F*^2^) = 0.080	*w* = 1/[σ^2^(*F*_o_^2^) + (0.0415*P*)^2^ + 0.2381*P*] where *P* = (*F*_o_^2^ + 2*F*_c_^2^)/3
*S* = 1.05	(Δ/σ)_max_ < 0.001
2589 reflections	Δρ_max_ = 0.12 e Å^−^^3^
168 parameters	Δρ_min_ = −0.20 e Å^−^^3^
0 restraints	Absolute structure: Flack (1983), 1077 Friedel pairs
Primary atom site location: structure-invariant direct methods	Flack parameter: −0.1 (3)

### Special details

**Table:**

Geometry. All e.s.d.'s (except the e.s.d. in the dihedral angle between two l.s. planes) are estimated using the full covariance matrix. The cell e.s.d.'s are taken into account individually in the estimation of e.s.d.'s in distances, angles and torsion angles; correlations between e.s.d.'s in cell parameters are only used when they are defined by crystal symmetry. An approximate (isotropic) treatment of cell e.s.d.'s is used for estimating e.s.d.'s involving l.s. planes.
Refinement. Refinement of *F*^2^ against ALL reflections. The weighted *R*-factor *wR* and goodness of fit *S* are based on *F*^2^, conventional *R*-factors *R* are based on *F*, with *F* set to zero for negative *F*^2^. The threshold expression of *F*^2^ > σ(*F*^2^) is used only for calculating *R*-factors(gt) *etc*. and is not relevant to the choice of reflections for refinement. *R*-factors based on *F*^2^ are statistically about twice as large as those based on *F*, and *R*- factors based on ALL data will be even larger.

### Fractional atomic coordinates and isotropic or equivalent isotropic displacement parameters (Å^2^)

**Table:**

	*x*	*y*	*z*	*U*_iso_*/*U*_eq_	
O1	0.78315 (11)	0.39621 (8)	0.41136 (8)	0.0206 (2)	
N1	0.76810 (12)	0.50948 (9)	0.20177 (9)	0.0152 (2)	
N2	0.55012 (12)	0.40406 (10)	0.18282 (10)	0.0161 (2)	
N3	0.52865 (13)	0.46113 (10)	0.08925 (10)	0.0176 (2)	
C1	0.91802 (15)	0.46592 (12)	0.18446 (12)	0.0180 (3)	
H1A	0.9139	0.3963	0.1401	0.022*	
H1B	0.9634	0.4482	0.2571	0.022*	
C2	1.01007 (16)	0.55293 (12)	0.12398 (12)	0.0212 (3)	
H2A	0.9713	0.5632	0.0479	0.025*	
H2B	1.1123	0.5258	0.1179	0.025*	
C3	1.00881 (16)	0.66502 (12)	0.18456 (13)	0.0221 (3)	
H3A	1.0614	0.6576	0.2561	0.027*	
H3B	1.0606	0.7216	0.1392	0.027*	
C4	0.85187 (16)	0.70368 (12)	0.20604 (13)	0.0219 (3)	
H4A	0.8531	0.7729	0.2510	0.026*	
H4B	0.8031	0.7205	0.1345	0.026*	
C5	0.76669 (16)	0.61340 (11)	0.26713 (12)	0.0201 (3)	
H5A	0.8116	0.5999	0.3408	0.024*	
H5B	0.6643	0.6382	0.2788	0.024*	
C6	0.66729 (15)	0.42747 (11)	0.24015 (11)	0.0159 (3)	
C7	0.68611 (15)	0.36763 (12)	0.34649 (11)	0.0172 (3)	
C8	0.58735 (18)	0.27024 (12)	0.37224 (12)	0.0236 (3)	
H8A	0.5897	0.2550	0.4522	0.035*	
H8B	0.6212	0.2041	0.3316	0.035*	
H8C	0.4868	0.2882	0.3497	0.035*	
C9	0.40458 (15)	0.43897 (11)	0.02405 (11)	0.0158 (3)	
C10	0.36359 (15)	0.51634 (12)	−0.05636 (11)	0.0177 (3)	
H10	0.4195	0.5825	−0.0668	0.021*	
C11	0.24038 (16)	0.49645 (13)	−0.12146 (11)	0.0215 (3)	
H11	0.2126	0.5490	−0.1768	0.026*	
C12	0.15788 (17)	0.40034 (14)	−0.10604 (12)	0.0246 (3)	
H12	0.0731	0.3875	−0.1500	0.030*	
C13	0.19957 (17)	0.32311 (13)	−0.02621 (13)	0.0249 (3)	
H13	0.1434	0.2570	−0.0160	0.030*	
C14	0.32279 (16)	0.34171 (12)	0.03888 (12)	0.0206 (3)	
H14	0.3512	0.2884	0.0933	0.025*	
H3	0.596 (2)	0.5100 (15)	0.0692 (16)	0.029 (5)*	

### Atomic displacement parameters (Å^2^)

**Table:**

	*U*^11^	*U*^22^	*U*^33^	*U*^12^	*U*^13^	*U*^23^
O1	0.0199 (5)	0.0240 (5)	0.0178 (5)	−0.0002 (4)	−0.0022 (4)	−0.0007 (4)
N1	0.0118 (5)	0.0150 (5)	0.0189 (5)	−0.0006 (4)	0.0002 (4)	−0.0004 (4)
N2	0.0153 (5)	0.0165 (5)	0.0166 (5)	0.0009 (5)	0.0010 (4)	−0.0002 (5)
N3	0.0141 (5)	0.0204 (6)	0.0184 (5)	−0.0045 (5)	−0.0016 (5)	0.0037 (5)
C1	0.0147 (6)	0.0190 (7)	0.0203 (6)	0.0023 (5)	0.0022 (6)	−0.0026 (6)
C2	0.0151 (6)	0.0244 (7)	0.0241 (7)	−0.0003 (6)	0.0043 (6)	0.0018 (6)
C3	0.0162 (7)	0.0221 (7)	0.0280 (7)	−0.0048 (6)	0.0004 (6)	0.0034 (6)
C4	0.0192 (7)	0.0167 (6)	0.0297 (7)	−0.0018 (6)	0.0014 (6)	0.0011 (6)
C5	0.0179 (7)	0.0167 (6)	0.0255 (7)	0.0009 (6)	0.0043 (6)	−0.0022 (6)
C6	0.0141 (6)	0.0156 (6)	0.0178 (6)	−0.0004 (5)	0.0020 (5)	−0.0018 (5)
C7	0.0170 (7)	0.0175 (7)	0.0171 (6)	0.0024 (5)	0.0017 (5)	−0.0029 (5)
C8	0.0299 (8)	0.0211 (7)	0.0199 (7)	−0.0047 (6)	−0.0008 (6)	0.0031 (6)
C9	0.0117 (6)	0.0187 (7)	0.0171 (6)	0.0002 (5)	0.0004 (5)	−0.0039 (5)
C10	0.0154 (6)	0.0203 (6)	0.0175 (6)	0.0000 (5)	0.0019 (5)	−0.0023 (5)
C11	0.0177 (7)	0.0286 (8)	0.0181 (6)	0.0044 (6)	−0.0001 (5)	−0.0019 (6)
C12	0.0156 (6)	0.0342 (8)	0.0241 (7)	−0.0019 (6)	−0.0052 (6)	−0.0072 (6)
C13	0.0192 (7)	0.0248 (7)	0.0307 (8)	−0.0061 (6)	−0.0009 (6)	−0.0047 (6)
C14	0.0180 (7)	0.0191 (7)	0.0247 (7)	−0.0034 (6)	−0.0014 (6)	−0.0006 (6)

### Geometric parameters (Å, °)

**Table:**

O1—C7	1.2288 (17)	C4—H4B	0.9900
N1—C6	1.4216 (17)	C5—H5A	0.9900
N1—C5	1.4712 (17)	C5—H5B	0.9900
N1—C1	1.4778 (17)	C6—C7	1.4767 (19)
N2—C6	1.3025 (18)	C7—C8	1.505 (2)
N2—N3	1.3317 (16)	C8—H8A	0.9800
N3—C9	1.4023 (17)	C8—H8B	0.9800
N3—H3	0.88 (2)	C8—H8C	0.9800
C1—C2	1.523 (2)	C9—C10	1.391 (2)
C1—H1A	0.9900	C9—C14	1.3934 (19)
C1—H1B	0.9900	C10—C11	1.390 (2)
C2—C3	1.526 (2)	C10—H10	0.9500
C2—H2A	0.9900	C11—C12	1.386 (2)
C2—H2B	0.9900	C11—H11	0.9500
C3—C4	1.526 (2)	C12—C13	1.386 (2)
C3—H3A	0.9900	C12—H12	0.9500
C3—H3B	0.9900	C13—C14	1.388 (2)
C4—C5	1.5201 (19)	C13—H13	0.9500
C4—H4A	0.9900	C14—H14	0.9500
			
C6—N1—C5	113.80 (10)	N1—C5—H5B	109.7
C6—N1—C1	113.63 (10)	C4—C5—H5B	109.7
C5—N1—C1	112.42 (10)	H5A—C5—H5B	108.2
C6—N2—N3	117.31 (11)	N2—C6—N1	120.44 (12)
N2—N3—C9	119.69 (11)	N2—C6—C7	116.78 (12)
N2—N3—H3	118.1 (13)	N1—C6—C7	122.75 (12)
C9—N3—H3	122.1 (13)	O1—C7—C6	119.99 (13)
N1—C1—C2	109.67 (11)	O1—C7—C8	121.03 (12)
N1—C1—H1A	109.7	C6—C7—C8	118.96 (12)
C2—C1—H1A	109.7	C7—C8—H8A	109.5
N1—C1—H1B	109.7	C7—C8—H8B	109.5
C2—C1—H1B	109.7	H8A—C8—H8B	109.5
H1A—C1—H1B	108.2	C7—C8—H8C	109.5
C1—C2—C3	111.58 (12)	H8A—C8—H8C	109.5
C1—C2—H2A	109.3	H8B—C8—H8C	109.5
C3—C2—H2A	109.3	C10—C9—C14	120.06 (13)
C1—C2—H2B	109.3	C10—C9—N3	118.74 (12)
C3—C2—H2B	109.3	C14—C9—N3	121.20 (13)
H2A—C2—H2B	108.0	C11—C10—C9	119.72 (13)
C4—C3—C2	110.75 (12)	C11—C10—H10	120.1
C4—C3—H3A	109.5	C9—C10—H10	120.1
C2—C3—H3A	109.5	C10—C11—C12	120.33 (14)
C4—C3—H3B	109.5	C10—C11—H11	119.8
C2—C3—H3B	109.5	C12—C11—H11	119.8
H3A—C3—H3B	108.1	C13—C12—C11	119.78 (13)
C5—C4—C3	110.24 (12)	C13—C12—H12	120.1
C5—C4—H4A	109.6	C11—C12—H12	120.1
C3—C4—H4A	109.6	C12—C13—C14	120.44 (14)
C5—C4—H4B	109.6	C12—C13—H13	119.8
C3—C4—H4B	109.6	C14—C13—H13	119.8
H4A—C4—H4B	108.1	C13—C14—C9	119.66 (14)
N1—C5—C4	109.69 (11)	C13—C14—H14	120.2
N1—C5—H5A	109.7	C9—C14—H14	120.2
C4—C5—H5A	109.7		
			
C6—N2—N3—C9	179.73 (12)	N2—C6—C7—O1	−170.31 (12)
C6—N1—C1—C2	169.70 (11)	N1—C6—C7—O1	7.84 (19)
C5—N1—C1—C2	−59.23 (15)	N2—C6—C7—C8	11.11 (18)
N1—C1—C2—C3	54.55 (15)	N1—C6—C7—C8	−170.74 (13)
C1—C2—C3—C4	−53.17 (16)	N2—N3—C9—C10	163.81 (12)
C2—C3—C4—C5	54.34 (16)	N2—N3—C9—C14	−16.06 (19)
C6—N1—C5—C4	−167.80 (11)	C14—C9—C10—C11	0.3 (2)
C1—N1—C5—C4	61.21 (15)	N3—C9—C10—C11	−179.57 (12)
C3—C4—C5—N1	−57.74 (16)	C9—C10—C11—C12	0.4 (2)
N3—N2—C6—N1	−1.12 (18)	C10—C11—C12—C13	−0.8 (2)
N3—N2—C6—C7	177.07 (11)	C11—C12—C13—C14	0.4 (2)
C5—N1—C6—N2	108.74 (14)	C12—C13—C14—C9	0.3 (2)
C1—N1—C6—N2	−120.87 (13)	C10—C9—C14—C13	−0.7 (2)
C5—N1—C6—C7	−69.34 (15)	N3—C9—C14—C13	179.22 (13)
C1—N1—C6—C7	61.04 (16)		

### Hydrogen-bond geometry (Å, °)

**Table:**

Cg1 is the centroid of the C9–C14 benzene ring.

**Table:**

*D*—H···*A*	*D*—H	H···*A*	*D*···*A*	*D*—H···*A*
N3—H3···O1^i^	0.88 (2)	2.47 (2)	3.2317 (15)	145.6 (17)
N3—H3···N1	0.88 (2)	2.242 (19)	2.6340 (16)	106.8 (15)
C2—H2b···Cg1^ii^	0.99	2.77	3.5535 (16)	137
C3—H3a···Cg1^iii^	0.99	2.98	3.9473 (16)	167

Symmetry codes: (i) −*x*+3/2, −*y*+1, *z*−1/2; (ii) *x*+1, *y*, *z*; (iii) −*x*+3/2, −*y*+1, *z*+1/2.
